# Epstein-Barr Virus-Negative Diffuse Large B-Cell Post-transplant Lymphoma in an Epstein-Barr Virus-Positive Recipient

**DOI:** 10.7759/cureus.18134

**Published:** 2021-09-20

**Authors:** Charlotte Lee, Helena Vincentelli, Jenni Visuri, Simon Knight, Rutger Ploeg

**Affiliations:** 1 Medical Sciences Division, University of Oxford, Oxford, GBR; 2 Nuffield Department of Surgical Sciences, University of Oxford, Oxford, GBR; 3 Oxford Transplant Centre, Oxford University Hospitals NHS Foundation Trust, Churchill Hospital, Oxford, GBR

**Keywords:** immunosuppression, small bowel resection, intussusception, pancreas and kidney transplant, ebv ptld

## Abstract

Post-transplant lymphoproliferative disease (PTLD) can arise as a complication after solid organ transplantation. Epstein-Barr virus (EBV) infection is a known risk factor and is known to drive disease manifestation. PTLD can occur in EBV-negative recipients and with EBV-negative donor organs, however, EBV-negative PTLD pathogenesis is unknown. Here, we present PTLD presenting as intussusception in a patient with a historic simultaneous pancreas-kidney transplant (SPK). This case study presents the first documented case of EBV-negative post-transplant lymphoproliferative disease in an EBV-seropositive SPK recipient from an EBV-positive donor. Here we describe the diagnosis and management of this patient, discuss the differences between EBV positive and negative driven post-transplant lymphoproliferative disease, and highlight areas of research opportunity in the latter.

## Introduction

Post-transplant lymphoproliferative disease (PTLD) encompasses a spectrum of mono- or poly-clonal lymphoid proliferations, from benign to malignant [1]. It is important to note that PTLD in solid organ transplant patients is usually of recipient origin, whereas hematopoietic stem cell transplantation (HSCT) related PTLD is usually of donor origin [1,2]. PTLD onset is classified as either early (within one year of transplant) or late (exceeding one year since transplant) [3]. Risk factors include the donated organ type, age of the recipient, and the use of tacrolimus and/or muromonab-CD3 (OKT3) [4]. There is no current estimation for the incidence of PTLD in simultaneous pancreas-kidney transplant (SPK) recipients.

PTLD can further be defined by Epstein-Barr virus (EBV) status. EBV-positive lymphomas tend to manifest early PTLD while EBV-negative lymphomas, late [1]. Approximately 60-80% of PTLD incidence is EBV driven. It is hypothesized that there are two distinct pathological processes leading to EBV-positive and EBV-negative post-transplant lymphomas, however, the etiology of EBV-negative PTLD is unknown [3,5]. Beyond PTLD classification by EBV status, the WHO classification is based on histological features of the disease and is widely used [6]. This system acknowledges the lymphoma cell lineage, the most common being of B-cell origin.

Here, we describe a 34-year-old immunosuppressed female patient presenting with a CT-diagnosed intussusception and a history of SPK (2014). PTLD was diagnosed following open small bowel resection. Both donor and recipient were seropositive for EBV infection prior to receipt of transplant.

## Case presentation

The patient was historically diagnosed with type 1 diabetes mellitus, with retinal and renal involvement. Subsequent acute renal failure in the context of pyelonephritis resulted in an SPK transplant from a deceased donor in 2014. Both the donor and recipient were EBV seropositive, tested via peripheral venous blood sampling. The transplant was complicated by duodenal segment breakdown and bile leak into the peritoneum. However, the patient experienced recovery after surgical revision and washouts. During recovery, the patient required an above-the-knee amputation due to left lower limb ischemia and infection following subsequent angioplasty. Immunosuppression was initially maintained with mycophenolate mofetil and tacrolimus. In 2019, the patient changed from mycophenolate mofetil to azathioprine in anticipation of pregnancy. Since transplantation, the patient was closely followed up and the transplanted organs had a good function. The patient’s EBV serostatus was monitored, demonstrating chronic EBV infection from pre-transplant to current presentation.

In 2019, the patient experienced episodes of diarrhea and nausea. Endoscopy did not visualize the transplant duodenal anastomosis, however, the surgeon noted two areas of non-bleeding angiodysplasia in the duodenum, which were cauterized by argon laser. The patient continued to have good functioning of the transplanted pancreas and kidney.

**Figure 1:**
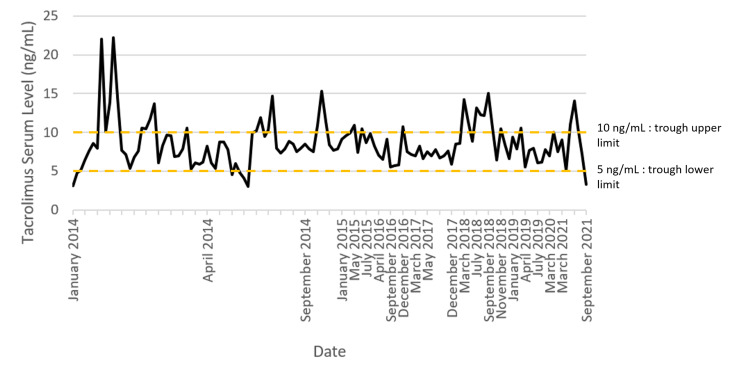
Patient serum tacrolimus levels (2014-2021). Trust trough serum range: 5-10 ng/mL.

In 2021, the patient was transferred to our service with CT-diagnosed intussusception. Tacrolimus serum levels were 14.1 ng/mL on admission (Figure 1). The patient underwent a tacrolimus-tapering regime in order to restore tacrolimus serum levels to within the trust’s recommended trough treatment range (5-10 ng/mL). The patient underwent an open small bowel resection in order to treat the intussusception. Due to historic abdominal surgeries, extensive adhesions were noted. Direct visualization of the intussusception located a mass, approximately 55 mm in maximum dimension, in the proximal jejunum approximately 30 cm proximal to the pancreatic enteric anastomosis. Interoperative appearances were consistent with small bowel lymphoma. In view of this lesion, surgical resection and end-to-end anastomosis were performed, with a margin of visible healthy bowel both proximally and distally. Palpable bowel lymphatic nodules were surgically resected. CT neck and thorax with contrast were performed and no cervical or thoracic lymphadenopathy was detected. Positron emission tomography imaging demonstrated no additional tumors. With complete resection, future planned management includes surveillance and close monitoring of tacrolimus serum levels. The patient was recently reviewed 2.5 weeks post-operation and demonstrated good recovery.

Histology and serology

On slicing, the cut surface was solid with a variegated appearance. Histological examination demonstrated a high-grade malignancy with areas of sheet-like necrosis. The tumor cells were large, pleomorphic lymphocytes with apoptotic debris and scattered mitoses. Large nucleoli were noted. The tumor proliferation index was high and estimated at up to 80%.

Immunostaining was positive for CD20, CD19, CD79a, Bcl-2, and Mum-1. C-myc was expressed in 60% of the tumor. In situ hybridization of the mass for EBV-encoded small RNAs (EBERs) was negative. The tumor was defined as an EBV-negative monomorphic PTLD, diffuse large B-cell lymphoma (non-germinal centre B-cell subtype).

## Discussion

Here, we present a case of EBV-negative small bowel lymphoma in an EBV-positive SPK recipient. There are two cases of EBV-negative PTLD occurring in EBV-positive HSCT patients, within a patient cohort of an epidemiological study [5]. The EBV serostatus of the hemopoietic stem cell donors was irretrievable [5]. However, here, we report that the patient received EBV-positive organs while demonstrating EBV seropositivity pre-transplant. This case demonstrates PTLD can arise through non-EBV-driven pathways, as here, EBV-negative PTLD arose in a chronically infected EBV host and EBV-positive donor organs.

Risk factors for PTLD include extremes of age, EBV infection (especially EBV infection post-transplant), HSCT, drug exposures, and genetic factors [4]. Broadly, it is thought that EBV-driven PTLD arises in immunosuppressed patients due to the inability to suppress EBV-driven B-cell proliferation, and/or impaired T-cell and natural killer (NK) cells immunosurveillance [5,7]. This can lead to B-cell immortalization. Recipients that are EBV-positive prior to transplant have an increased risk for PTLD [4], however, this risk is significantly lower than EBV-negative recipients receiving EBV-positive organs [8]. It is concluded that PTLD manifestation in this patient was not EBV driven, as in situ hybridization studies demonstrated the lesion to be EBV negative. The mechanism of non-EBV-driven PTLD pathogenesis remains undefined [7].

The patient was also positive for the risk factor of tacrolimus exposure; their immunosuppression was maintained with tacrolimus since their SPK transplant in 2014 (Figure 1). Tacrolimus is known to inhibit T-cell function and thus decrease T-cell immunosurveillance of aberrant B-cells [9]. Large cohort trials have demonstrated an association between tacrolimus exposure and a significantly shorter time to PTLD development [4,8]. It should be noted that these studies did not record PTLD lesion's EBV status. The role of tacrolimus exposure in the development of EBV-negative PTLD is unknown. Here, the patient’s tacrolimus serum level was poorly controlled (Figure 1). The patient exceeded the trust’s target trough level (5-10 ng/mL) sporadically, however, not significantly since 2018. It should be noted that the levels were monitored every three months, with a two-month delay in 2020 due to the coronavirus pandemic. As such, we consider that tacrolimus may have contributed to a pro-PTLD environment with sporadic over-exposure events. However, the data collection intervals are too broad to confidently propose tacrolimus as a direct contributing factor to PTLD development.

The patient’s PTLD was specifically defined as a non-germinal center B-cell subtype. Literature suggests non-germinal center subtype lymphoma is associated with a worse prognosis in immunocompetent patients [10]. However, outcomes of immunosuppressed patients have not been widely reported. Further, determining if the PTLD was of donor or recipient origin would offer completeness. As the patient received a solid organ transplant and there were no primary tumors detected, the lesion is most likely of recipient origin [2].

## Conclusions

In summary, we present the first documented case of EBV-negative post-transplant lymphoproliferative disease in an EBV-seropositive SPK recipient from an EBV-positive donor. This case highlights the lack of definition of non-EBV-driven PTLD pathways, therefore, the need for research in this area. Here, we consider sporadic over-exposure to tacrolimus as a contributor to fostering a pro-PTLD environment. Therefore, we encourage strict control of tacrolimus serum levels, for example, through an increase of serum level monitoring.
